# Dentate gyrus progenitor cell proliferation after the onset of spontaneous seizures in the tetanus toxin model of temporal lobe epilepsy

**DOI:** 10.1016/j.nbd.2013.02.001

**Published:** 2013-06

**Authors:** Premysl Jiruska, Anan B.Y. Shtaya, David M.S. Bodansky, Wei-Chih Chang, William P. Gray, John G.R. Jefferys

**Affiliations:** a Neuronal Networks Group, School of Clinical and Experimental Medicine, University of Birmingham, Birmingham B15 2TT, UK; b Department of Developmental Epileptology, Institute of Physiology, Academy of Sciences of Czech Republic, Prague, CZ-14220, Czech Republic; c Department of Neurology, Charles University, 2nd School of Medicine, University Hospital Motol, Prague, CZ-15006, Czech Republic; d Wessex Neurological Centre, Southampton University Hospital Trust, Southampton, SO16 6YD, UK; e School of Medicine (Clinical Neurosciences), Faculty of Medicine, Health and Life Sciences, University of Southampton, Southampton, SO16 6YD, UK; f National Institute of Neuroscience and Mental Health Research, LGF — Henry Wellcome Building, Heath Park, Cardiff, CF14 4XN, UK

**Keywords:** Spontaneous seizures, Temporal lobe epilepsy, Neurogenesis, Tetanus toxin, Apoptosis

## Abstract

Temporal lobe epilepsy alters adult neurogenesis. Existing experimental evidence is mainly from chronic models induced by an initial prolonged status epilepticus associated with substantial cell death. In these models, neurogenesis increases after status epilepticus. To test whether status epilepticus is necessary for this increase, we examined precursor cell proliferation and neurogenesis after the onset of spontaneous seizures in a model of temporal lobe epilepsy induced by unilateral intrahippocampal injection of tetanus toxin, which does not cause status or, in most cases, detectable neuronal loss. We found a 4.5 times increase in BrdU labeling (estimating precursor cells proliferating during the 2nd week after injection of toxin and surviving at least up to 7 days) in dentate gyri of both injected and contralateral hippocampi of epileptic rats. Radiotelemetry revealed that the rats experienced 112 ± 24 seizures, lasting 88 ± 11 s each, over a period of 8.6 ± 1.3 days from the first electrographic seizure. On the first day of seizures, their duration was a median of 103 s, and the median interictal period was 23 min, confirming the absence of experimentally defined status epilepticus. The total increase in cell proliferation/survival was due to significant population expansions of: radial glial-like precursor cells (type I; 7.2 ×), non-radial type II/III neural precursors in the dentate gyrus stem cell niche (5.6 ×), and doublecortin-expressing neuroblasts (5.1 ×). We conclude that repeated spontaneous brief temporal lobe seizures are sufficient to promote increased hippocampal neurogenesis in the absence of status epilepticus.

## Highlights

► Progenitor cell proliferation and neurogenesis increase in tetanus toxin TLE. ► Repeated spontaneous brief temporal lobe seizures are sufficient to promote neurogenesis. ► Neurogenesis and progenitor cell proliferation occur without status epilepticus.

## Introduction

The generation of new neurons, neurogenesis, in the adult brain continues in mammals, including humans, throughout life (Eriksson et al., 1998; Gage et al., 1998). Neurogenesis is regulated by a variety of physiological stimuli and newly born dentate gyrus neurons integrate into the circuitry of the adult hippocampus, leading to theories for roles in learning and memory (Deng et al., 2010).

In animal models, hippocampal neurogenesis is affected by a wide range of pathological conditions including of focal epilepsies, in particular those induced by initial status epilepticus (Bender et al., 2003; Bengzon et al., 1997; Gray and Sundstrom, 1998; Jessberger et al., 2005, 2007; Nakagawa et al., 2000; Parent et al., 1997). Despite the difficulties in assessing neurogenesis in adult humans, where robust controlled methods for detection cannot be implemented ethically, evidence from surgically excised human tissue supports the presence of altered neurogenesis in clinical temporal lobe epilepsy (TLE) (Blumcke et al., 2001; Crespel et al., 2005; Fahrner et al., 2007; Parent et al., 2006) especially in patients with poor memory performance preoperatively (Coras et al., 2010). In animal models, which allow the detection of changes with a greater temporal fidelity, hippocampal neurogenesis is initially enhanced following the induction of status epilepticus (Bengzon et al., 1997; Gray and Sundstrom, 1998; Jessberger et al., 2005, 2007; Parent et al., 1997) and later declines in some (Hattiangady and Shetty, 2008) but not all models (Bonde et al., 2006). However, while previous studies have examined the differential effects of severe convulsive versus less severe status epilepticus on hippocampal neurogenesis (Bouilleret et al., 1999; Hung et al., 2012; Mohapel et al., 2004; Yang et al., 2008), there have been no studies to our knowledge examining the effects of repeated spontaneous seizures without a prior status. The fully convulsive status epilepticus based models of TLE used previously (induced by kainate, pilocarpine or electrical stimulation) are typically associated with substantial morphological changes, in the form of neuronal loss and sprouting of axonal collaterals. Few studies have demonstrated seizure-induced cell proliferation and/or neurogenesis in the absence of status epilepticus. Bengzon et al. (1997) found single evoked afterdischarges could increase neurogenesis 3-fold and a rapid kindling protocol (40 stimuli at 5 min) increased it 6-fold, in both cases with a concomitant increases in apoptosis (TUNEL staining) of newly generated neurons/precursor cells. Smith et al. (2005) found increased neurogenesis for at least 21 days (respectively) following rapid amygdala kindling, without neuronal loss, in contrast with Bengzon et al. (1997). Conventional kindling, with daily or twice-daily stimulation, increased neurogenesis for several days following stage 5 seizures, but not in the earlier stages of kindling or after single afterdischarges (Nakagawa et al., 2000; Scott et al., 1998, 2010). Ferland et al. (2002) observed a transient increase in BrdU labeled cells 3 days after a single flurothyl seizure or up to 7 days after 8 daily seizures, in this case without evidence of neuronal death. Single febrile seizures at P10 did not induce increased neurogenesis, although more prolonged seizures induced by kainic acid did (Bender et al., 2003). However, none of these paradigms can model the effect of spontaneous onset seizures on neurogenesis of potential relevance to the 60% of patients with TLE in whom there is no antecedent history of prolonged febrile seizures (Waruiru and Appleton, 2004).

Intrahippocampal tetanus toxin induces epilepsy with spontaneous and recurrent seizures but without major morphological changes (Jefferys et al., 1992; Jiruska et al., 2010; Mellanby et al., 1977), and notably without status epilepticus at any stage (Finnerty et al., 2000; Hawkins and Mellanby, 1987; Jiruska et al., 2010). While the majority of rats gain seizure remission in this model after 6–8 weeks, they retain abnormal cellular pathophysiology (Vreugdenhil et al., 2002), permanent cognitive and other behavioral impairments (Brace et al., 1985; Mellanby, 1982), and a minority continues to seize (Mellanby, 1993). This model provides a means of testing the effect of early repeated spontaneous seizures on cell proliferation and neurogenesis independently of prolonged status epilepticus.

## Materials and methods

### Animals

Sixteen adult male Sprague-Dawley rats weighing approximately 250 g were housed under standard conditions in a room with controlled temperature (22 ± 1 °C) and 12/12 h light/dark cycle. The animals had ad libitum access to food and water. All animal procedures were licensed and performed in strict accordance with the Animal Scientific Procedures Act (1986) of the United Kingdom and with Institutional Ethical Review.

### Surgery, recording and BrdU injections

Surgical preparation was performed under ketamine/methibromide or isoflurane anesthesia. Small trephine openings were drilled symmetrically over both hippocampi at coordinates 4.1 mm caudal to bregma and 3.9 mm either side of the midline using the atlas of Paxinos and Watson (1998). Using a Hamilton microsyringe and infusion pump (KD Scientific Inc., USA) 1 μl of tetanus toxin (Sigma-Aldrich, UK) solution was injected into the stratum radiatum of the right hippocampal CA3 area. Tetanus toxin solution contained 25 ng of tetanus toxin in 1 μl of 0.05 M phosphate buffered saline (PBS; Sigma-Aldrich, UK) and 2% bovine serum albumin (Sigma-Aldrich, UK). Tetanus neurotoxin solution was injected at 200 nl/min. The microsyringe was left in hippocampus for 5 min after the injection ended to avoid the solution leaking back through the injection track. Control animals were injected with 1 μl of 0.05 M PBS with 2% bovine serum albumin. Following the injections, silver ball electrodes were inserted into both openings epidurally over both cortices and fixed to the skull using dental acrylic. Electrodes were connected to single channel bipolar telemetric transmitters (Data Sciences International, s'Hertogenbosch, Netherlands) which were implanted subcutaneously over the dorsal aspect of the thorax and secured with sutures. Following surgery, animals were housed in single cages and allowed to recover for two days. Continuous synchronized video-electrocorticography monitoring started on the 4th day, which precedes the onset of spontaneous seizures (Jefferys and Walker, 2006) and continued until the end of the experiments. Electrocotricography was recorded using Dataquest A.R.T. 4.3 aquisition system (Data Sciences International, s'Hertogenbosch, Netherlands) and sampled at 100 Hz. Video was recorded synchronously using digital infra-red cameras (Y-cam Solutions Ltd, Richmond, UK) and Spike2 software (Cambridge Electronic Design, Cambridge, UK). Recorded signals were exported, reviewed and analyzed using Spike2, to verify the development of spontaneous seizures and to determine seizure frequency and duration. Seizures were classified as secondary generalized when they progressed into falls followed by generalized convulsions.

On day 10 (while still being continuously monitored), rats received the first of seven daily intraperitoneal injections of bromodeoxyuridine solution (BrdU, 50 mg/kg, 10 mg/ml in 0.007 M NaOH/0.9% sterile saline; Sigma-Aldrich, UK). On day 17, 24 h after the last BrdU injection, animals were humanely overdosed with ketamine and then perfused using 0.9% saline followed by 4% paraformaldehyde. Brains were extracted and postfixed in 4% paraformaldehyde.

### Immunohistochemistry

The brains were sectioned along the coronal plane at 40 μm intervals through the entire hippocampus on a vibratome (Leica VT1000M, Leica Microsystems Ltd, Milton Keynes, UK). Each slice was transferred to an individual well (of a 24 well plate) containing PBS for storage. All immunohistochemistry was performed on systematically sampled tissue, with the initial section selected randomly and subsequent sections being taken at constant intervals thereafter, ensuring the entire dentate gyrus was sampled. BrdU immunostaining was performed on 12 sections per animal. Caspase-3 labeling and doublecortin (Dcx) labeling were performed on 6 systematically sampled sections from each animal. For double stain immunohistochemistry of BrdU and Dcx, sections were incubated in 2 M HCl at 37 °C for 30 min, followed by washing and incubation with 3% H_2_O_2_–10% methanol for 30 min. Saturation of non-specific binding sites was achieved in 5% donkey serum in 0.25% Triton X-100 (1 h). This was followed by overnight incubation of primary antibodies to BrdU (rat monoclonal 1:1000; Oxford Biotech, UK) and Dcx (goat polyclonal 1:200; Santa Cruz, CA, USA). The final step was incubation with secondary antibodies Alexa 448/594 raised in donkey (1/500, Invitrogen).

Rabbit polyclonal cleaved caspase-3 primary antibody was used at 1:200 (New England Biolabs, Hitchen, UK). Triple immunohistochemistry studies for BrdU, Sox2 (1:500 Goat polyclonal Santa Cruz Biotechnology, CA, USA) and anti-rabbit GFAP (1/500, DAKO) were also conducted to look at the proliferation of both type I and type II/III precursor cells in subgranular zone (Encinas et al., 2006). Secondary antibodies were Alexa 488, Alexa 594, and Alexa 647 (1:500, Invitrogen, Life Technologies Ltd, Paisley, UK) all were raised in donkey and matched the primary combinations. Finally the slices were mounted on glass microscope slides with Mowiol mounting medium (Harlow Chemicals, Harlow, Essex, UK) and stored in the dark at 4 °C to delay damage from exposure to ultraviolet light. Non-specific secondary antibody binding was excluded by the lack of immunostaining in control experiments omitting the primary antibody.

### Cell quantification

A blind counting methodology was employed for all quantification. Masks of the subgranular zone and granule cell layer were generated with the contour tool in StereoInvestigator™ (Ver. 5.0; Microbrightfield Inc., Williston, VT) software package on each dentate gyrus. Counting of cells utilized the StereoInvestigator system (Microbrightfield Inc.) interfaced through a Dialux 22 Leitz microscope equipped with a color camera (Optronics Inc., Muskogee, OK). Using a 100 × oil immersion lens, the BrdU positive cells (BrdU +) were counted from 60 to 400 randomly and systematically selected frames (each measuring 40 × 40 μm, 0.0016 mm^2^ area) in every 12th section. In brief, the contour of the subgranular zone regions was marked (two cell layer zone at the border of the granule cell layer and hilus) (Hattiangady et al., 2004) in every section through the tracing function of the StereoInvestigator. The optical fractionator component was then selected and the number and location of counting frames and the counting depth for that section were ascertained via entering parameters such as the grid size, the thickness of the top guard zone (4 μm) and the optical dissector height (8 μm). A computer driven motorized stage then facilitated the section to be analyzed at each of the counting frame locations. All BrdU + cells that were present within the 8 μm section depths in each location were counted.

The StereoInvestigator program then calculated the total number of BrdU + cells per subgranular zone by the optical fractionator formula:

$$N=\frac{1}{\mathrm{ssf}}\cdot \frac{1}{\mathrm{asf}}\cdot \frac{1}{\mathrm{hsf}}\cdot \mathrm{EQ}2$$

where *ssf* represents the section sampling fraction, which was 12 in this study as every 12th section was sampled; *asf* symbolizes the area sampling fraction, which is calculated by dividing the area sampled with the total area of the subgranular zone (i.e., the sum of subgranular zone areas sampled in every 12th section); *hsf* stands for the height sampling fraction, which is calculated by dividing the height sampled (i.e., 8 μm in this study) with the section thickness at the time of analysis (i.e., 20–25 μm); *EQ2* denotes the total count of particles sampled for the entire DG. Ipsilateral and contralateral BrdU + cells were counted in all animals.

For triple immunohistochemistry (Sox2, GFAP, and BrdU), 100 BrdU + cells were counted from at least 4 representative sections per brain. The proportion of BrdU + cells that expresses Sox2, GFAP or both was measured: the image from each stain was obtained sequentially, taking care to avoid bleed-through between the emission spectra for the respective fluorophores. All imaging was performed on a Leica SP5 laser scanning confocal microscope (Leica DMI600 inverted microscope frame), generating Z-stacks of 1 μm per image; all three-dimensional images were analyzed.

### Statistical analysis

Results were statistically analyzed using SPSS (IBM Software Group, IBM Corporation Armonk, USA) and GraphPad Prism (GraphPad Software, Inc., La Jolla, CA) software. The following statistical tests were used: t-test, one-way ANOVA with post hoc comparisons, Mann–Whitney, Kolmogorov–Smirnov, and Pearson correlation. All results are shown as mean ± S.E.M. unless otherwise stated.

## Results

### Spontaneous recurrent seizures after tetanus toxin injection

**Fig. 1:**
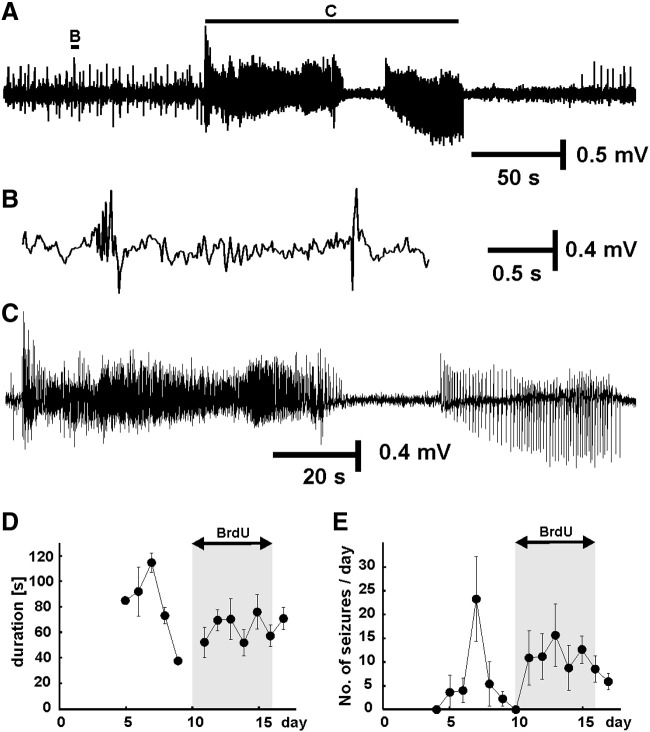
Electrographic epileptic activity and seizure profile. A) Example of electrocorticographic recording from the epileptic animal, characterized by interictal discharges (B) and spontaneous seizures (C). D) Graph showing temporal profile of mean seizure duration (8 animals). Gray area marks days during which BrdU was injected. E) Temporal profile of mean seizure frequency (8 animals).

Repeated spontaneous seizures started between 5 and 14 days after tetanus toxin injection (median 6 days). Seizures were classified as complex partial seizures characterized by behavioral arrest, staring and oroalimentary or sniffing automatisms. These seizures progressed into unilateral or bilateral forelimb jerks, rearing, falling and secondary generalized tonic–clonic seizures. Seizures usually ended with repeated wet-dog shakes. Electrocorticogram activity between seizures was characterized by spikes or sharp-wave discharges (Fig. 1B), and during seizures by prolonged rhythmic discharges (Fig. 1C).

Radiotelemetry revealed that the rats experienced 112 ± 24 seizures in total and 76 ± 14 during the BrdU application (Figs. 1D,E). Average seizure frequency was 11.4 ± 2.4 day^− 1^ over the whole experimental protocol and 9.8 ± 2 day^− 1^ during the BrdU application. Mean seizure duration was 88.0 ± 10.6 s during the whole protocol and 78.3 ± 10.8 s during BrdU administration period. Total duration of seizure activity recorded throughout the protocol was 9439 ± 1841 s, and 5703 ± 1356 s during BrdU application. A distinguishing feature of the tetanus toxin model is that it is not characterized by an initial episode of experimental status epilepticus (Cavalheiro et al., 2006; Dudek et al., 2006; Turski et al., 1987). To check whether that was the case in this series of rats we measured all the seizures on the first day they appeared and found that the median between-seizure interval was 23.3 min and median of seizure duration was 103 s. The lack of behavioral status in this model has been extensively documented (reviewed in Jefferys and Walker, 2006), and evident from routine post-operative care following every injection. In a separate study we performed long-term telemetry recordings of hippocampal activity starting within minutes of injecting tetanus toxin into the hippocampus and found that there was no status epilepticus during the first 3 days, covering the gap at the start of the recordings reported here (W-C Chang and J G R Jefferys, unpublished data), and consistent with previous work (Hawkins and Mellanby, 1987).

### Spontaneous recurrent seizures increase dentate gyrus cell proliferation/survival in the tetanus toxin model of TLE

**Fig. 2:**
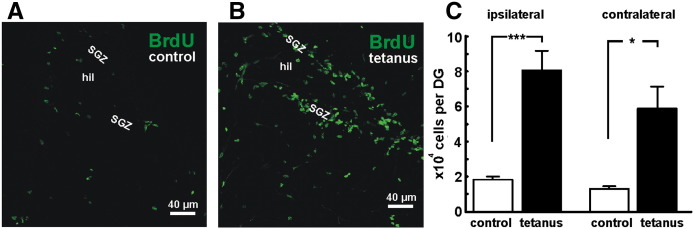
Increase in dentate gyrus cellular BrdU incorporation in the tetanus toxin model of epilepsy. A) Immunohistochemical detection of BrdU + cells in ipsilateral dentate gyrus of control animal. B) Increased number of BrdU + cells in dentate gyrus of epileptic animals. BrdU + cells are located mainly in subgranular zone and granule cell layer. Several cells are also dispersed in hilus of the dentate gyrus. C) Counts of BrdU positive cells in dentate gyrus of the injected (“ipsilateral”) hippocampi show significantly more BrdU incorporating cells in epileptic rats (***p < 0.001; 8 animals in each group). Significant difference in BrdU + cell count was also observed dentate gyrus contralateral to the injection (*p < 0.05; 5 animals in each group).

Immunohistochemistry demonstrated significantly more cells incorporating BrdU in animals with recurrent spontaneous seizures (Fig. 2). Our BrdU labeling paradigm reflects a combination of both cell proliferation and subsequent survival at the time of killing. Ipsilateral to the toxin injection the dentate gyrus contained a mean of 80,532 ± 11,052 BrdU + cells (8 rats), while in control animals it contained 18,292 ± 1658 cells (8 rats), demonstrating a 4.4 times greater number of BrdU + cells in epileptic animals than in controls (p < 0.001; Mann–Whitney test). Contralateral to the injection the dentate gyrus contained 4.5 times more BrdU + cells in epileptic rats (58,604 ± 12,506; 5 rats) than in controls (13,095 ± 1521; 4 rats; p < 0.05; Mann–Whitney test). There were no significant differences in numbers of BrdU + cells between injected and contralateral sides in either the tetanus toxin or control animals. The majority of BrdU + cells were located in the subgranular zone of dentate gyrus (Fig. 2B).

### Spontaneous recurrent seizures increase the proliferation of both type I and type II/III precursor cells in the adult DG

**Fig. 3:**
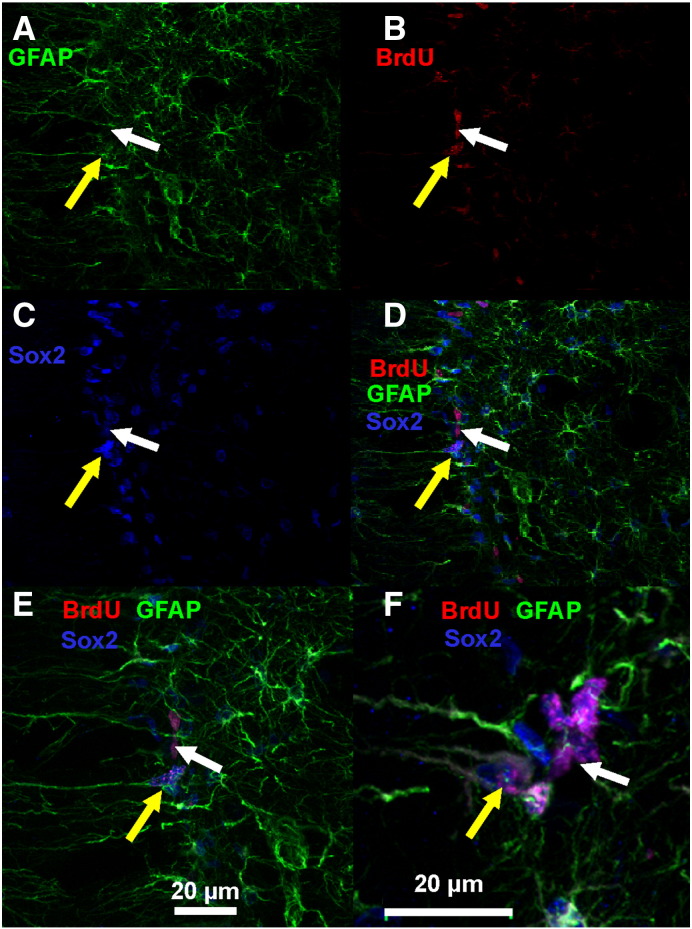
Increased precursor cell proliferation and survival in the adult DG after seizures. A) GFAP labeling. B) BrdU immunolabeling. C) Sox2 immunostaining. D) Combined triple immunolabeling identifies type I precursor cells (yellow arrows) and type II/III precursors (white arrow) in epileptic dentate gyrus. E) Detail of type I and type II/III precursors. F) Detail of triple labeling in different slices with examples of each precursor subtypes.

Having demonstrated an increase in cellular BrdU incorporation after recurrent spontaneous seizures, we determined the phenotype of the BrdU positive cells in the dentate gyrus. We identified a significant increase in the number of type I precursor cells incorporating BrdU (expressing Sox2, GFAP and BrdU) with a radial glial-like morphology (Fig. 3). In control animals the total number of type I precursors was 2117 ± 368 cells (4 rats), increasing in epileptic animals by 7.2 times to 15,860 ± 2267 (Fig. 5; 3 rats; p < 0.05, Mann–Whitney test). Moreover, the proliferation and subsequent survival of type II/III precursors, expressing Sox2 but not GFAP (Fig. 3), and thus classified as amplifying neural progenitors and neuroblasts, also increased significantly from 8119 ± 1372 cells/DG in control conditions (4 rats) to 45,860 ± 6555 cells/DG in epileptic animals (Fig. 5; 3 rats; p < 0.05; Mann–Whitney test).

### Neurogenesis is increased in the tetanus toxin model of TLE

**Fig. 4:**
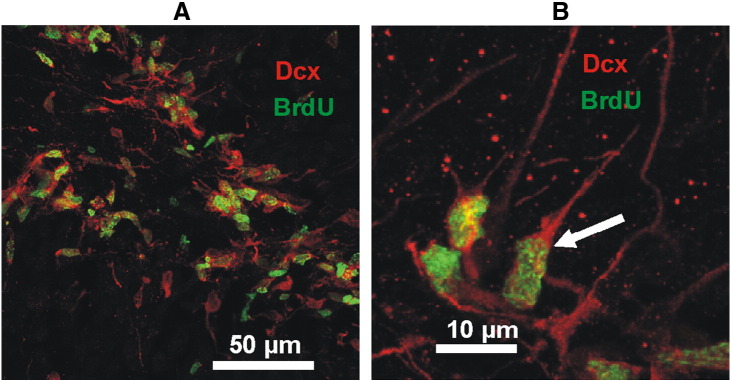
Neurogenesis in the adult DG after seizures. A,B) Doublecortin positive cells (Dcx, red) that incorporated BrdU (green) in the subgranular zone of an epileptic animal at low (A) and higher magnification (B; arrow).

**Fig. 5:**
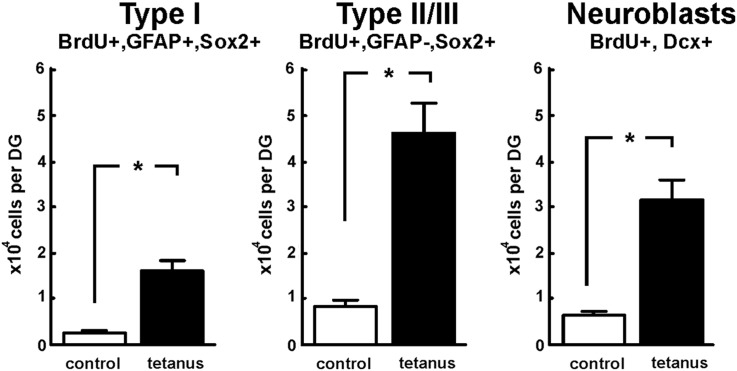
Quantification of precursor cell proliferation/survival and neurogenesis. The number of BrdU + type I, type II/III increased significantly in animals with spontaneous recurrent seizures (*p < 0.05). Cell counts of Dcx + and BrdU + positive cells demonstrate significant increase in their number in epileptic dentate gyrus.

The next question was whether spontaneous recurrent seizures affect newly born neurons (neuroblasts) and thus neurogenesis. Using double immunohistochemistry for doublecortin (Dcx, immature neuronal marker) and BrdU (Fig. 4), we demonstrated that total number of BrdU-labeled immature neurons and neuronally committed precursors was 5.1 times greater in epileptic dentate gyrus (31,080 ± 4443; 3 rats) than in controls (Fig. 5; 6078 ± 1027, 4 rats; p < 0.05; Mann–Whitney test). Neither proliferation nor neurogenesis was significantly correlated with measures of seizure duration or frequency.

### Activated caspase-3 immunostaining in the dentate gyrus is not increased 17 days after toxin injection

It was shown previously that hippocampal sclerosis is present only in 10% of animals and affects mainly CA1 (Jefferys et al., 1992; Jiruska et al., 2010). To examine whether apoptotic cell death occurred in the present study, sections from the ipsilateral hippocampi from five epileptic animals (six sections from each animal) and four control animals were stained for caspase-3. Caspase-3 is a member of the cysteine–aspartic acid protease (caspase) family, activation of which plays a central role in the execution-phase of cell apoptosis. We found no difference in the number of activated caspase-3 positive cells between epileptic (1524 ± 92 cells; 5 rats) and control animals (1545 ± 143 cells; 4 rats), at the time of killing.

## Discussion

The central results from this study are that the spontaneously-seizing tetanus toxin model of temporal lobe epilepsy in the absence of status epilepticus results in a ~ 5-fold increase in BrdU labeled cells in both the injected and contralateral hippocampi during the second week after induction. Increases in neurogenesis (Dcx + cells) and in type I (with radial glial-like morphology) and type II/III (amplifying neural progenitors) precursor cells matched those in BrdU labeling. Previous work using lesional chronic models of TLE showed that neurogenesis is altered in epilepsy in ways that depend on the severity of the initial insult or injury that causes epilepsy, i.e. convulsive or non-convulsive status epilepticus (Hung et al., 2012; Mohapel et al., 2004; Yang et al., 2008), the time after the initial status epilepticus (Bonde et al., 2006; Cha et al., 2004; Hattiangady et al., 2004) and on the model used.

It has been suggested that one of the mechanisms up-regulating neurogenesis in TLE is neuronal cell loss (Kuruba et al., 2009), and given that the relationship between status epilepticus and neuronal loss is well documented, this provides a potential reason for the increased neurogenesis in chronic epilepsies induced by status epilepticus. Kindling-type stimulations have little or no impact on neuronal numbers, however an early study showing increased neurogenesis after repeated afterdischarges evoked by rapidly-repeated kindling-type stimulations was associated with evidence of neuronal precursor death (Bengzon et al., 1997), although other reports of conventional kindling describe increased neurogenesis during later (after several stage 5 seizures) and not earlier stages (Nakagawa et al., 2000; Scott et al., 1998, 2010). The chronic epilepsy induced by tetanus toxin used in the present study is not associated with status epilepticus, as confirmed in the present study by quantification of the first day's seizures, which revealed brief seizures separated by relatively long interictal periods, and is categorically different from experimental status epilepticus (Cavalheiro et al., 2006; Dudek et al., 2006). We found no increases in activated caspase-3 positive cells, consistent with previous studies on the tetanus toxin model which showed absence of neuronal loss in the majority (~ 90%) of rats (Jefferys et al., 1992; see also Discussion in Jiruska et al., 2010), or bilateral selective loss of about one third of hilar somatostatin-positive neurons, but not evident until 8 weeks after injection of tetanus toxin (Mitchell et al., 1995).

Several studies have dissociated seizure-related neurogenesis from neuronal death using models of symptomatic seizures, induced by flurothyl (Ferland et al., 2002). Single electrically-evoked afterdischarges (Bengzon et al., 1997) led to both the proliferation and apoptosis of neuronal precursors and differentiated neurons, which contrasts with our repeated spontaneous seizures which cause neurogenesis without increased activated caspase-3 staining. However, our examination of apoptosis was late, and does not at all exclude the possibility of increased apoptosis of precursors or newly born granule cell neurons at the time of the initial spontaneous seizures, which would have been cleared by the time of death. Rapid kindling stimulation in the hippocampus led to greater increases in both apoptosis and neurogenesis. Amygdalar kindling in mice and rats (Nakagawa et al., 2000; Scott et al., 1998, 2010; Smith et al., 2005) demonstrated the expected chronic reductions in seizure threshold without spontaneous recurrent seizures, and led to transiently increased neurogenesis, peaking at 2–7 days and subsequently disappearing, without evidence of neuronal death.

In addition to the increased number of immature neurons, we observed increases in precursor cells (Bonaguidi et al., 2011; Encinas et al., 2006; Kempermann, 2011), including type I (radial glial) precursors. This is consistent with previous reports of increased proliferation of this cell type after status epilepticus (Huttmann et al., 2003; Steiner et al., 2008). This significant increase may suggest either self renewal of this sub-population of cells, due to symmetric cell proliferation, and/or recruitment from a quiescent pool (Bonaguidi et al., 2011). Cells that expressed Sox 2 but not GFAP (type II/III precursors) increased more than five times in response to spontaneous recurrent seizures. In addition, the numbers of BrdU positive doublecortin positive cells increased more than five times in this tetanus toxin model. Thus, more neuronal precursors proliferate and subsequently survive across all sub groups.

Our study shows that precursor cell dynamics and short-term neurogenesis are altered after the onset of spontaneous temporal lobe seizures in a manner not dissimilar to that after status epilepticus. The demonstration here of altered short-term neurogenesis after the onset of spontaneous seizures may be relevant to the 60% of patients who present with TLE without a history of status, suggesting that their hippocampal neurogenesis may be altered by initial spontaneous seizures. Whether or not this alteration in neurogenesis after the onset of spontaneous seizures is long-lasting remains to be investigated, but if it is, it may be relevant to long-term cognitive impairment which is found in the intrahippocampal tetanus toxin model (Brace et al., 1985; Mellanby et al., 1982). Long-term cognitive impairments are associated with abnormal human neurogenesis under in-vitro conditions (Coras et al., 2010; Kempermann et al, 2004), and pharmacological strategies to reverse learning and memory deficits in TLE may operate by modulating neurogenesis (Barkas et al., 2012).
